# Cannabis use in patients treated for opioid use disorder pre- and post-recreational cannabis legalization in Canada

**DOI:** 10.1186/s13011-021-00372-z

**Published:** 2021-04-13

**Authors:** Tea Rosic, Nitika Sanger, Balpreet Panesar, Gary Foster, David C. Marsh, Launette Rieb, Lehana Thabane, Andrew Worster, Zainab Samaan

**Affiliations:** 1 Department of Psychiatry and Behavioural Neurosciences, McMaster University, 1280 Main St W, Hamilton, ON L8S 4L8 Canada; 2 Department of Health Research Methods, Evidence, and Impact, McMaster University, 1280 Main St W, Hamilton, ON L8S 4L8 Canada; 3 Medical Sciences Graduate Program, McMaster University, 1280 Main St W, Hamilton, ON L8S 4L8 Canada; 4 Neurosciences Graduate Program, McMaster University, 1280 Main St W, Hamilton, ON L8S 4L8 Canada; 5 Biostatistics Unit, Research Institute at St Joseph’s Healthcare, 50 Charlton Avenue East, Hamilton, ON L8N 4A6 Canada; 6 Northern Ontario School of Medicine, 935 Ramsey Lake Rd, Sudbury, ON P3E 2C6 Canada; 7 Canadian Addiction Treatment Centres, 175 Commerce Valley Drive West, Suite 300, Markham, Ontario L3T 7P6 Canada; 8 ICES North, 41 Ramsey Lake Road Sudbury, Sudbury, ON P3E 5J1 Canada; 9 Department of Family Practice, University of British Columbia and St. Paul’s Hospital, 1081 Burrard St, Hornby site, Vancouver, BC V6Z 1Y6 Canada; 10 Department of Medicine, McMaster University, 1280 Main St W, Hamilton, ON L8S 4L8 Canada

**Keywords:** Legalization, Cannabis, Comorbidity, Polysubstance use, Recreational, Methadone, Buprenorphine

## Abstract

**Background:**

As the legalization of recreational cannabis becomes more widespread, its impact on individuals with substance use disorders must be studied. Amidst an ongoing opioid crisis, Canada’s legalization of recreational cannabis in October 2018 provides an important setting for investigation. We examined changes to cannabis use patterns in patients receiving medication-assisted treatment (MAT) for opioid use disorder (OUD) following legalization.

**Methods:**

This study includes cross-sectional data from 602 participants recruited 6 months pre-legalization and 788 participants recruited 6 months post-legalization, providing information on cannabis use. Regression analysis was used to estimate the association between legalization and cannabis use patterns. We collected longitudinal urine drug screens (UDSs) detecting cannabis-metabolites for 199 participants recruited pre-legalization and followed prospectively post-legalization. Conditional logistic regression was used to assess the association between legalization and UDS results.

**Results:**

Past-month cannabis use was self-reported by 54.8 and 52.3% of participants recruited pre- and post-legalization, respectively. Legalization was not associated with changes in any measured cannabis characteristics: cannabis use (OR 0.91, 95% CI 0.73–1.13), days of use/month (*B* -0.42, 95% CI - 2.05-1.21), money spent, or cannabis source. There was no association between legalization and prevalence of cannabis use on UDS (OR 1.67, 95% CI 0.93–2.99) or percentage of cannabis-positive UDSs (OR 1.00, 95% CI 0.99–1.01). Participants overwhelmingly reported that legalization would have no impact on their cannabis use (85.7%).

**Conclusions:**

Amongst patients treated for OUD, no significant change in cannabis use was observed following legalization; however, high rates of cannabis use are noted.

## Background

Cannabis remains one of the most commonly used substances worldwide [1]. As such, jurisdictions around the world deliberate the legalization of its recreational use. In Canada, the prevalence of cannabis use was approximately 12% in adults prior to legalization (2015 data) [2]. Cannabis use was expected to increase among adults in the general population following the legalization of recreational cannabis on October 17, 2018, as evidenced by increases in cannabis use in several American jurisdictions following legalization [3, 4]. Indeed, preliminary Canadian data indicate that cannabis use has increased particularly among individuals older than 25 years (from 13.1 to 15.5%) between 2018 and 2019 [5].

The impact of cannabis legalization policies on individuals with psychiatric disorders, including substance use disorders, is unknown and warrants specific attention. Prior to legalization, individuals with psychiatric disorders have been demonstrated to have higher prevalence of cannabis use and cannabis use disorder than the general population, a finding that persists when controlling for sociodemographic factors [6]. This phenomenon has been attributed to self-medication of psychiatric symptoms [7], common biological and psychosocial risk factors [8], and the risk of subsequent development of psychiatric disorders in the context of cannabis use [6, 9]. In addition to higher rates of cannabis use, the most commonly cited adverse effects of cannabis including anxiety, impaired cognition, and psychosis are more likely to impact those with comorbid mental illness and addiction [10].

Further still, the current cannabis policy changes have taken place amidst an ongoing opioid crisis in North America that has not abated despite efforts to alter the trajectory of the crisis [11, 12]. We have previously found that 51% of participants enrolled in a cohort study of medication-assisted treatment (MAT) for OUD use cannabis [13], while 28% meet criteria for cannabis use disorder [14] – rates that are significantly higher than in the general population [2]. Cannabis has been explored for its harm reduction potential as a substitute drug in the treatment of other substance use disorders, including opioids, alcohol and benzodiazepines [15, 16]. However, findings from a systematic review of 23 studies on this topic found no consensus on opioid use or treatment retention in the context of cannabis use during methadone maintenance treatment [17]. Similarly, the relationship between cannabis laws and trends in the opioid crisis has been a major topic of study. Results are mixed and researchers highlight the need for caution in their interpretation: cannabis laws have been associated with reduced prescription opioid use [18], and reductions in opioid overdoses [19], yet other evidence suggests cannabis laws are not associated with reductions in opioid overdose mortality [20].

The higher pre-legalization prevalence of cannabis use in individuals with psychiatric disorders, and the ongoing acuity of the opioid crisis, make understanding the impact of policy changes on individuals with OUD critical. Attention has been paid, primarily, to the impact of cannabis and its legalization, on opioid use. In this study, we shift the focus and examine trends related to the use of cannabis itself within a population of patients with OUD, pre- and post-legalization:

1. Are there differences in patients’ self-reported cannabis use patterns pre- and post-legalization of recreational cannabis in Canada?
2. Was there an increase in cannabis use, as measured by urine drug screens, in the 6 months following legalization?
3. Do patients report an impact of legalization on their cannabis use?

## Methods

### Data

**Fig. 1:**
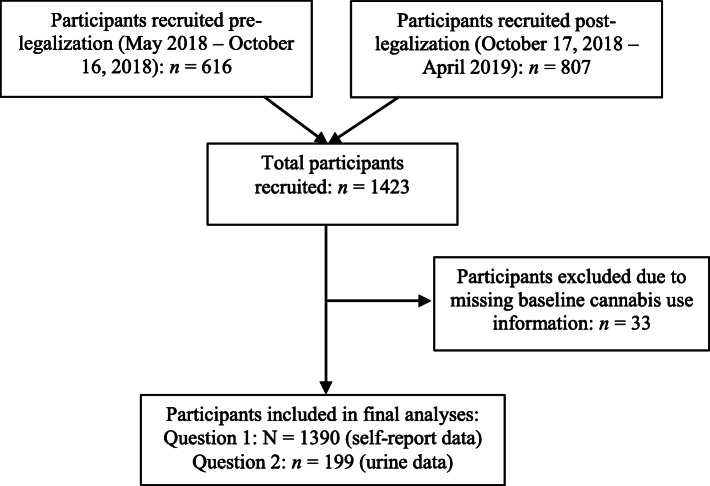
Study Flow Diagram

We used data collected in the Pharmacogenetics of Opioid Substitution Treatment Response (POST) study. The POST study aimed to examine genetic and psychosocial factors, including cannabis legalization, associated with outcomes in MAT. Recruitment began in May 2018 from 27 outpatient MAT clinics in Ontario, Canada. For the purposes of examining the impact of cannabis legalization, the present study includes participants recruited in the 6-month period prior to legalization (May – October 2018), and participants recruited in the 6-month period following legalization (October – April 2019). Study inclusion criteria were: males and females aged 16 years or older, diagnosed with OUD as per the American Psychiatric Association’s *Diagnostic and Statistical Manual of Mental Disorders, Fifth Edition* (DSM-5) criteria and receiving MAT with methadone or buprenorphine-naloxone. No other inclusion or exclusion criteria were applied in order to increase the generalizability of this study; therefore, participants with any comorbid mental health diagnoses were included. Participants could be enrolled in treatment for any length of time prior to recruitment and were approached by study personnel and recruited consecutively as they attended previously scheduled clinic appointments. Altogether, 602 participants were recruited in the pre-legalization group, and 788 participants were recruited in the post-legalization group. Ethics approval was obtained from the Hamilton Integrated Research Ethics Board (project ID 4556). Written informed consent was obtained from all participants. Participants were excluded from the main analyses of this study if they failed to self-report whether or not they used cannabis (*n* = 33; Fig. 1). This study is reported in accordance with the Strengthening the Reporting of Observational Studies in Epidemiology (STROBE) guidelines [21].

### Study instruments and measures

All participants completed cross-sectional assessments at study entry including sociodemographic information, MAT history including medication, dose, time in treatment, and cannabis use information. Self-reported cannabis use in the last 30 days was assessed using the Maudsley Addiction Profile (MAP) [22]. We previously reported the sensitivity and specificity of self-report of cannabis use was 79.9% (95% confidence interval [CI] 72.7–85.8) and 80.0% (95% CI 73.6–85.4) in the OUD population [13]. The Comprehensive Marijuana Motives Questionnaire (CMMQ) [23] was also administered to provide more information on patterns and motivations for cannabis use. The CMMQ is a 36-item self-report measure representing 12 different motives for using marijuana: enjoyment, conformity, coping, experimentation, boredom, alcohol use, celebration, altered perceptions, social anxiety, relative low risk, sleep, and availability [23]. The CMMQ was validated in a non-OUD population and not in the context of harm reduction, drug substitution, or for chronic pain [23]. Therefore, we augmented the CMMQ to address pain relief, appetite stimulation, drug substitution, relief of opioid withdrawal symptoms, relief of opioid craving, and relief of cannabis withdrawal symptoms, tailored to the OUD population. We also asked all participants questions about the cost, source, and form of cannabis used in the last month. Finally, all participants responded to the open-ended question, “Will/did legalization of recreational cannabis impact your use?”

At the time of the study intake, all participants were asked the open-ended question “Will/did legalization of recreational cannabis impact your use”. The inclusion of open-ended questions in the cross-sectional study entry interview was added as an exploratory component to complement the quantitative findings of this study. All consecutively recruited participants were asked the same open-ended questions and there was no selective participant criteria or sampling strategy employed when collecting qualitative data. Research staff conducting these interviews have backgrounds in addiction research and had previously participated in another study investigating factors associated with methadone treatment outcomes [24]; however, interviewers were not known to the participants of the research study. The interviewers previous experience in administering study interviews at these addiction clinics facilitated their familiarity with terminology used by study participants, which assisted in transcription of answers to open-ended questions. Verbal responses provided by study participants were transcribed verbatim in online anonymized records.

Participants were followed in this study prospectively for 12 months. Urine drug screens were administered approximately weekly, following routine clinical protocol, and tested for opioids and opioid metabolites using the FaStep Assay (Trimedic Supply Network Ltd., Concord, Ontario, Canada) [25]. Urine screening for cannabis metabolites was not included in the routine UDS panel at all participating clinics; however, for a subgroup of participants in the pre-legalization group (*n* = 199/602) who attended clinics in which cannabis metabolites were tested, these results are available longitudinally, covering both pre- and post-legalization periods. Pre-legalization drug screens were assessed for up to 12 months before study entry, and post-legalization drug screens were assessed for 6 to 12 months post-study entry to ensure that these results were from the post-legalization period. We present the demographic and clinical characteristics of participants who received UDSs for cannabis metabolites compared to the total pre-legalization group in Appendix 1.

### Analysis

We conducted statistical analyses using STATA version 15.1 (StataCorp LP, College Station, TX, USA). We summarized continuous variables using means with standard deviation (SD) or medians with interquartile range (IQR) for normally distributed and skewed data, respectively. We summarized categorical variables using frequencies and percentages. We compared cross-sectional data on cannabis use patterns between the pre-legalization group and the post-legalization group. Pairwise comparisons were made using two-sample t-tests for continuous variables, chi-squared tests for dichotomous variables, and analysis of variance tests for multichotomous variables, with results reported using *p* values. In order to estimate the association between cannabis legalization and cannabis use characteristics, we constructed regression models in which the dependent variable was the cannabis use characteristic, and legalization group status was a covariate. All estimates were adjusted for participant age (patients older than 25 report higher cannabis use) [5], sex (males report more cannabis use than females) [13], medication (buprenorphine-naloxone or methadone), medication dose (higher doses are associated with lower substance use) [26, 27], length of time in treatment (polysubstance use is associated with worse retention in treatment) [28], and opioid use (abstinence or ongoing use; opioid use is associated with other substance use during treatment) [29], based on clinical rationale for their association with cannabis use and OUD treatment outcomes. All covariates were entered into the model simultaneously and no covariates were excluded on the basis of statistical testing due to a priori clinical hypotheses related to their relevance. For dichotomous outcome variables we present estimates of association as odds ratios (ORs) with 95% CI and *p* values, and for continuous outcomes we present unstandardized beta-coefficients with 95% CI and *p* values. To note, the use of regression analysis to estimate the association between legalization and cannabis use patterns allows for adjustment for important aforementioned covariates; the use of univariate tests such as t-tests, ANOVAs, and chi-square tests do not allow for such adjustment. For the subgroup of pre-legalization participants for whom longitudinal UDSs were available (*n* = 199), we used conditional logistic regression analysis to estimate the association between cannabis legalization and cannabis-positive UDSs, conditioned on the participant, where each participant acts as their own control. Results are presented as ORs with 95% CI and *p* values.

The qualitative approach used to analyze responses to the open-ended question: “Will/did legalization of recreational cannabis impact your use?” was a data-driven thematic analysis [30] which we conducted using Nvivo 12 Qualitative Data analysis software (QSR International [Americas] Inc., Burlington, Massachusetts, USA). Before importing the data into Nvivo, we began by familiarizing ourselves with the responses provided by the study participants. We actively read, and re-read responses, minimizing typographical errors as we went through each data entry. After importing text pertaining to “recreational cannabis use” as an open-ended question we began to classify main ideas, key phrases and patterns into nodes using the coding software. We ran open-ended data through word and text frequency queries to determine the most common words and their stemmed variants in order to assist us in the coding of data into nodes. We used multiple word frequency queries in order to avoid decontextualization of the answers included in the open-ended questions. We paired the queries with manual coding of the responses and gave each data item equal attention. The coding process resulted in the generation of a codebook. The next phase consisted of altering the codebook by collapsing similar nodes and labelling some pertinent and distinct nodes as themes. The final phase consisted of the re-working of themes to ensure all patterns are coherent, followed by the refinement of themes. Refinement of themes allowed for reflection of the coding process and coded data and assisted in the development of theme names [30, 31].

Descriptive statistics were used to separate the themes that were generated into subgroups of pre-legalization and post-legalization to complement the subgroups created when presenting the quantitative results. Specific responses from participants when asked about the impact of legalization on their cannabis use were used to provide support for discussion points and theories drawn from the quantitative results.

## Results

### Demographic and clinical characteristics

We included 1390 unique participants in the final analyses: 602 in the pre-legalization group and 788 in the post-legalization group (Fig. 1). The majority of participants in both groups were male (57 and 60%, respectively), and the mean age was 38.9 years (SD = 10.4) and 39.6 years (SD = 10.8), respectively. Most participants were receiving treatment with methadone (81% methadone versus 19% buprenorphine, in the total study sample, Table 1). There were no significant demographic or clinical differences between the groups including MAT medication doses, length of time in treatment, or ongoing opioid use (Table 1).

**Table 1:** Baseline demographic and clinical characteristics for patients recruited pre- and post- recreational cannabis legalization (*N* = 1390)

Characteristic	Total Sample (*N* = 1390)	Recruited pre-legalization (*n* = 602)	Recruited post-legalization (*n* = 788)	***p*** value
Age in years; mean (SD)	39.3 (10.7)	38.9 (10.4)	39.6 (10.8)	0.295
Male sex; *n* (%)	817 (58%)	343 (57%)	474 (60.2%)	0.222
Type of MAT; *n* (%)				0.073
Methadone	1120 (80.7%)	498 (82.9%)	622 (79%)	
Buprenorphine	268 (19.3%)	103 (17.1%)	165 (21%)	
Dose (mg/day); median (IQR)				
Methadone	68 (64)	65 (61)	70 (64)	0.276
Buprenorphine	12 (9.5)	12 (8)	10 (10)	0.097
Length of time in treatment (years); median (IQR)	2 (5.25)	2 (4.9)	2.3 (3.5)	0.756
Opioid abstinence^a^; *n* (%)	417 (30%)	176 (29.2%)	241 (30.6%)	0.587
Percentage of opioid-positive urine drug screens amongst non-abstainers^a^; mean (SD)	22.6 (23.6)	21.1 (23.3)	23.7 (23.8)	0.090

Participant baseline demographic and clinical characteristics

*SD* standard deviation, *MAT* medication-assisted treatment, *IQR* interquartile range

^a^Based on all urine drug screens collected up to 12 months pre-study entry

### Cannabis use characteristics, pre- and post- recreational cannabis legalization

Cannabis use was self-reported by 54.8 and 52.3% of participants recruited pre- and post-legalization, respectively (Table 2). The majority of cannabis users in both groups reported daily use (65.5% versus 67.2%). The most commonly identified reason for cannabis use was for “relaxation” in both groups (43.7% pre-legalization versus 41.2% post-legalization). Few participants identified that their reason for cannabis use was to manage cannabis withdrawal or cravings (6% in total study sample). Nearly 30% of participants in both groups reported using cannabis for pain relief.

**Table 2:** Self-reported cannabis use characteristics, for patients recruited pre- and post- recreational cannabis legalization (*N* = 1390)

Characteristic	Total Sample (*N* = 1390)	Recruited pre-legalization (*n* = 602)	Recruited post-legalization (*n* = 788)	***p*** value
Self-reported cannabis use; *n* (%)	742 (53.4%)	330 (54.8%)	412 (52.3%)	0.348
Days of use in the last 30 days amongst users; median (IQR)	30 (20)	30 (16)	30 (20)	0.540
Frequency of use; n (%)				0.896
Daily	492 (66.4%)	216 (65.5%)	276 (67.2%)	
Every other day	41 (5.5%)	21 (6.4%)	20 (4.9%)	
2–3 times per week	91 (12.3%)	45 (13.6%)	46 (11.2%)	
Once weekly	32 (4.3%)	12 (3.6%)	20 (4.9%)	
2–3 times per month	85 (11.5%)	36 (10.9%)	49 (11.9%)	
Dollars spent on cannabis per week (CAD); median (IQR)	25 (60)	30 (65)	20 (50)	0.023
Source of cannabis; *n* (%)				0.932
Dispensary	123 (16.6%)	51 (15.5%)	72 (17.5%)	
Medical Prescription	30 (4.1%)	17 (5.2%)	13 (3.2%)	
Vape Shop	16 (2.2%)	12 (3.6%)	4 (1%)	
Family/friends	276 (37.3%)	109 (33%)	167 (40.6%)	
Street	196 (26.5%)	103 (31.2%)	93 (22.6%)	
Other	100 (13.5%)	38 (11.5%)	62 (15.1%)	
Route of cannabis use; *n* (%)				0.708
Inhaled	527 (71.2%)	237 (71.8%)	290 (70.4%)	
Oral Ingestion	53 (7.1%)	21 (6.4%)	32 (7.8%)	
Oil	45 (6.1%)	19 (5.8%)	26 (6.3%)	
Other	117 (15.8%)	53 (16.1%)	64 (15.5%)	
Reasons for cannabis use^a^; *n* (%)				
To get high	297 (21.4%)	128 (21.3%)	169 (21.5%)	
Relaxation	588 (42.3%)	263 (43.7%)	325 (41.2%)	
Pain relief	400 (28.8%)	179 (29.7%)	221 (28.1%)	
Prescribed medication	74 (5.3%)	44 (7.3%)	30 (3.8%)	
Appetite stimulation	335 (24.1%)	154 (25.6%)	181 (23%)	
Peer pressure	142 (10.2%)	58 (9.6%)	84 (10.7%)	
Pleasure	358 (25.8%)	176 (29.2%)	182 (23.1%)	
Stress relief	516 (37.1%)	223 (37%)	293 (37.2%)	
Boredom	285 (20.5%)	126 (20.9%)	159 (20.2%)	
Social anxiety relief	284 (20.4%)	138 (22.9%)	146 (18.5%)	
Sleep promotion	503 (36.2%)	230 (38.2%)	273 (34.6%)	
Instead of opioids	287 (20.7%)	133 (22.1%)	154 (19.5%)	
Opioid withdrawal/craving relief	243 (17.5%)	108 (17.9%)	135 (17.1%)	
Cannabis withdrawal/craving relief	83 (6%)	36 (6%)	47 (6%)	

Self-reported cannabis use characteristics

*IQR* interquartile range, *CAD* Canadian Dollars

^a^Self-reported reasons for cannabis use; participants could indicate more than one reason

Cannabis legalization was found not to be significantly associated with self-reported cannabis use (OR 0.91, 95% CI 0.73–1.13; Table 3) or number of days of use in the last 30 days (*B* -0.42, 95% CI -2.05-1.21). Similarly, cannabis legalization was not associated with dollars spent on cannabis per week, source of cannabis, or route of use (Table 3).

**Table 3:** Estimates of association between cannabis legalization and self-reported cannabis use characteristics (N = 1390)

Characteristic	Estimate of association with cannabis legalization^**a**^	95% CI	***p***
Self-reported cannabis use	OR = 0.91	0.73, 1.13	0.409
Days of use in the last 30 days amongst users	*B* = −0.42	-2.05, 1.21	0.615
Daily frequency of use (daily versus other)	OR = 0.97	0.77, 1.21	0.794
Dollars spent on cannabis per week (CAD)	*B* = −6.41	−17.2, 4.39	0.244
Source of cannabis (dispensary or medical prescription versus other)	OR = 1.05	0.75, 1.48	0.780
Route of use (inhaled versus other)	OR = 0.93	0.68, 1.29	0.678

Association between cannabis legalization and self-reported cannabis use characteristics

*OR* odds ratio, *B* unstandardized beta-coefficient, *CI* confidence interval, *CAD* Canadian Dollar

^a^All estimates adjusted for age, sex, medication, dose, length of time in treatment, and illicit opioid abstinence

### Urine drug screen results

For a subgroup of 199 participants in the pre-legalization group (33% of the group), UDS results for cannabis metabolites were available for up to 12 months pre-study entry (pre-legalization) and for 6 to 12 months post-study entry (post-legalization; Table 4). Although there was no known systematic difference between clinics that administered UDS for cannabis metabolites and those that did not, we examined demographic and clinical differences between these groups in order to understand any potential differences (Appendix 1). There was a lower proportion of males in the group of participants who were followed with UDSs for cannabis metabolites compared to the total pre-legalization group (48.7% versus 57%). Additionally, participants in clinics completing cannabis UDSs were, on average, in treatment for longer (median 3 years (IQR = 5) versus median 2 years (IQR = 5.3)). Self-reported cannabis use was comparable (54.8 and 55.8%; Appendix 1).

**Table 4:** Subgroup analysis: Estimates of association between cannabis legalization and urine cannabis drug screen results (*n* = 199)

Urine drug screen results^**a**^	Pre-legalization statistic	Post-legalization statistic	Estimate of association with cannabis legalization	95% CI	***p***
Cannabis user	n = 139 (69.9%)	*n* = 135 (67.8%)	OR = 1.67	0.93, 2.99	0.087
Percentage of cannabis-metabolite-positive drug screens	Median = 75 IQR = 100	Median = 71.4 IQR = 100	OR = 1.00	0.99, 1.01	0.638

Subgroup analysis: Estimates of association between cannabis legalization and urine cannabis drug screen results

*CI* confidence interval, *OR* odds ratio, *IQR* interquartile range

^a^Urine drug screens for cannabis-metabolites were available for a subgroup of participants who were recruited pre-legalization (*n* = 199). Pre-legalization drug screens were assessed for up to 12 months before study entry, and post-legalization drug screens were assessed for 6 to 12 months post-study entry, ensuring that these results were from the post-legalization period

Within the subgroup of participants who had cannabis UDSs, 69.9% were identified as cannabis-users based on at least one positive UDS pre-legalization, and 67.8% were identified as cannabis-users based on at least one positive UDS post-legalization. Altogether, 108 participants had cannabis-positive UDSs in both periods, 45 participants had no cannabis-positive UDSs at both time periods, and 27 participants had new cannabis-positive UDSs and 19 participants stopped having cannabis-positive UDSs (data not shown). Using conditional logistic regression analysis, we found a non-significant association between legalization and prevalence of cannabis users in this subgroup (OR = 1.67, 95% CI 0.93–2.99). Furthermore, the median percentage of cannabis-positive UDSs in this subgroup was 75 and 71.4% in the pre- and post-legalization periods, respectively. We also found a non-significant association between legalization and percentage of cannabis-positive UDSs (OR = 1.00, 95% CI 0.99–1.01).

### Perceptions of legalization impacting cannabis use

The themes generated after conducting qualitative analysis were “No impact on use”, “Increase of use”, and “Decrease of use”. Prior to legalization, the majority of participants reported that legalization of recreational cannabis would have no impact on their use (85.9%), while 6.8% of participants indicated that legalization would increase their use and 4.2% reported that legalization would decrease their use (Appendix 2). Post-legalization, most participants similarly reported that legalization had no impact on their use (85.7%), while 4.7% reported that legalization increased their use, and 8.6% reported it decreased their use (Appendix 2). Participants who stated that legalization will increase their use commented “Yes, I will use more of it” and “Yes, I am more likely to smoke marijuana because it’s more accessible”, whereas participants who said that it will decrease their use commented that “I’ll use less”, “I will use it less because I sell it and the demand is high and I don’t have any left” and “I will use it less because it is more readily available so there is no urge to go and find it”. Participants who stated that legalization will have no impact their use provided comments such as “There will be no impact on use”, “No, I will use the same amount” and “No, I’ll use the same amount. I can just get it easier”.

## Discussion

In this observational study, we examined the impact of recreational cannabis legalization on cannabis use in individuals receiving treatment for OUD and found no significant differences in prevalence or patterns of cannabis use following legalization. More than 50% of participants self-reported cannabis use, and of those, the majority reported daily use. This is consistent with previously documented rates of cannabis use in patients with OUD, ranging from 40 to 75% [13, 32, 33]. Our findings suggest that for patients with an existing substance use disorder, many of whom already use cannabis, the legalization of recreational cannabis has not changed cannabis use behaviors. These findings were consistent with patients’ self-reported perceptions that legalization would have no impact on their cannabis use. The results from our cross-sectional analyses were supported using prospective analysis of cannabis-positive UDSs in a subgroup of participants. Our findings contribute to the growing literature on the impact of cannabis legalization as these policy changes take effect in jurisdictions worldwide.

Our finding of no significant change in self-report or UDS-confirmed cannabis use post-legalization may have numerous explanations. Individuals with OUD frequently have a long history of contact with the illicit (non-medical) drug market and have purchased their supplies in this fashion for years; legalization may be less likely to impact these patterns of drug use. Some participants had a “cannabis for medical purposes” exemption prior to legalization, thus were already using cannabis legally, and did not change their patterns post-legalization for recreational use. This is supported by a response recorded from a participant who stated “There will be no impact on my use. I use medical marijuana”, when asked if legalization will impact their use. Furthermore, in the year leading up to recreational cannabis legalization, cannabis use became a de facto law (i.e., people knew they would not be arrested or prosecuted for possession), thus use patterns may have risen and plateaued prior to legalization without significant impact of a change in the law. In Canada, prices of legal cannabis vary between provinces and have fluctuated between being higher and lower than illicit sources, possibly disincentivizing a change in supplier [34]. Prices of cannabis in Ontario during the pre- and post-legalization period captured in our study were $7.42 CAD per gram and $8.05 CAD per gram, respectively [34]. Our relatively short study timeframe, 6 months pre-legalization and 6-months post-legalization, also carries limitations. The complexity of the process by which legalization unfolded in Canada also lends possible explanation to our findings. Recreational cannabis became legal on October 17, 2018; however, access has been limited and varied [35]. The contrast between the participant responses of “It made it harder to get weed” and “No, I’ll use the same amount. I can just get it easier”, when asked about the impact of legalization on their use, highlights this variability in access. More studies will be required to continue to examine the impacts of this policy change for patients with OUD and other substance use disorders over longer periods of time. A more comprehensive understanding of risks or benefits of cannabis use in patients with OUD is also required.

While we found no significant changes in cannabis use due to legalization in this study, this may not be the case in other high-risk or vulnerable populations and these findings should not be extrapolated beyond the population under investigation here. The National Cannabis Survey conducted quarterly by Statistics Canada examines changes to patterns of cannabis use in the general population [34]. After legalization, in the first quarter of 2019, 18% of Canadians aged 15 years and older reported using cannabis in the past 3 months, compared to 14% in the first quarter of 2018 (prior to legalization) [34]. A significant decrease was found for accessing cannabis from the illegal market or from friends and family following legalization [34], although 42% of respondents still reported purchasing at least some of their cannabis from illegal sources and 37% reported using cannabis obtained from family and friends [34]. There is evidence to suggest increased risk for cannabis use disorder in the general population following recreational cannabis legalization [3].

In light of the substantial toll of the opioid crisis, scientists, clinicians, and policymakers must thoughtfully and consistently examine the influence of political and social factors on outcomes borne by patients affected by OUD. This study does just that, with a focus on understanding the impact of cannabis legalization specifically in individuals with OUD. The lack of an initial increase in the prevalence of cannabis use following legalization may be regarded as reassuring, however long-term impacts of this policy change have yet to be elucidated.

This is the first study, to our knowledge, to investigate the impact of this policy change on patients with OUD. It is unclear, however, whether our findings generalize to settings outside of Ontario, Canada, specifically in jurisdictions where MAT for OUD follows an abstinence-based approach such that patients with polysubstance use (including cannabis) are discharged from treatment. Furthermore, the outcomes of legalization in different countries around the world may be variable. Reports on the US experience of legalizing recreational cannabis use indicate that legalization has reduced the price of cannabis, increased its potency, and has led to increased use amongst adults [36]. Our study is limited by its primarily cross-sectional design (for self-report data), comparing two separate groups of participants recruited pre-legalization and post-legalization rather than a single group followed prospectively. The present analyses represent a secondary analysis of data collected for the POST study; therefore, power calculations were not conducted for this specific research question. Finding no statistically significant difference in cannabis use may be explained by low statistical power. Our augmented version of the CMMQ, including questions relevant to the OUD population, was not formally validated.

## Conclusions

This study contributes to an early examination of the impact of legalization of recreational cannabis on patients receiving treatment for OUD – an ongoing public health crisis in Canada and throughout the world. We identified no significant differences in cannabis use patterns pre- and post-legalization of recreational cannabis. As the post-legalization landscape in jurisdictions worldwide continues to evolve, future studies will be required to further examine the long-term impacts of legalization and outcomes in both opioid and cannabis use.

## Data Availability

The datasets used and/or analysed during the current study are available from the corresponding author on reasonable request.

## Appendix 1

**Table 5:** Comparison of demographic and clinical characteristics of participants recruited pre-legalization with and without cannabis urine screen data

Characteristic	Total pre-legalization sample (*n* = 602)	Pre-legalization sample with cannabis urine drug screens followed through post-legalization period (*n* = 199)
Age in years; mean (SD)	38.9 (10.4)	40.6 (11.1)
Male sex; *n* (%)	343 (57)	97 (48.7)
Type of MAT; *n* (%)		
→ Methadone	498 (82.9)	171 (85.9)
→ Buprenorphine	103 (17.1)	28 (14.1)
Dose (mg/day); median (IQR)		
→ Methadone	65 (61)	75 (63)
→ Buprenorphine	12 (8)	12 (7)
Length of time in treatment (years); median (IQR)	2 (4.9)	3 (5)
Opioid abstinence^a^; *n* (%)	176 (29.2)	66 (33.2)
Percentage of opioid-positive urine drug screens amongst non-abstainers^a^; mean (SD)	21.1 (23.3)	16.5 (18.7)
Self-reported cannabis use; *n* (%)	330 (54.8%)	111 (55.8)
Days of use in the last 30 days amongst users; median (IQR)	30 (16)	30 (15)

Characteristics of participants recruited pre-legalization with and without cannabis urine screen data

*SD* standard deviation, *IQR* interquartile range

## Appendix 2

**Table 6:** Qualitative analysis: “Will/did legalization of recreational cannabis impact your use?”

	Themes	***n*** (%)
**Pre-legalization**	It will have no impact	517 (85.9%)
	It will have an impact	
	• Will increase use	41 (6.8%)
	• Will decrease use	25 (4.2%)
	• No indication of increase or decrease	13 (2.2%)
**Post-legalization**	It had no impact	675 (85.7%)
	It did have an impact	
	• Increased use	45 (5.7%)
	• Decreased use	68 (8.6%)
	• No indication of increase or decrease	2 (0.3%)

Qualitative analysis results

## Abbreviations

OUD: Opioid use disorder
MAT: Medication assisted treatment
CMMQ: Comprehensive marijuana motives questionnaire
UDS: Urine drug screen
SD: Standard deviation
IQR: Interquartile range
OR: Odds ratio
CI: Confidence interval
